# Florence Nightingale's Contemporary Legacy for Patient Safety: Implications for Nursing and Public Health

**DOI:** 10.1111/nin.70149

**Published:** 2026-08-02

**Authors:** Elaine Lazaro Alcantara, Camila Pureza Guimarães da Silva, Antonio José de Almeida Filho, Hanna Carolina Neto Cavalcanti, Sabrina da Costa Machado Duarte, Tânia Cristina Franco Santos

**Affiliations:** ^1^ Facultad de Ciencias de la Salud Universidad Católica Santo Toribio de Mogrovejo Chiclayo Lambayeque Peru; ^2^ Anna Nery School of Nursing Federal University of Rio de Janeiro Rio de Janeiro Rio de Janeiro Brazil

**Keywords:** health policy, history of nursing, nursing theory, patient safety, public health nursing, quality of care

## Abstract

This paper critically examines Florence Nightingale's legacy and its ongoing relevance to contemporary patient safety. Drawing on *Notes on Nursing*, the study highlights Nightingale's emphasis on hygiene, cleanliness, environmental factors, and systematic care as foundational to reducing harm and improving patient outcomes. Her insights laid the groundwork for infection prevention and control practices that remain central to modern nursing and healthcare safety protocols. Nightingale's principles align closely with current patient safety frameworks and global initiatives, such as the World Health Organization's Global Action Plan, emphasizing the social responsibility of healthcare professionals. Despite scientific advances and evolving healthcare systems, her legacy continues to influence nursing practices by reinforcing systematized, ethical, and patient‐centered care. This enduring relevance underscores the critical role of nursing in safeguarding health and promoting safety in diverse clinical settings. This study advocates for ongoing recognition and integration of Nightingale's principles to enhance patient safety and care quality in modern healthcare. Her legacy remains an epistemological and practical resource for advancing nursing theory, patient‐centered care, and critical reflection in healthcare.

**Trial Registration:** Not applicable

## 1 Introduction

Patient safety has been a central aspect of healthcare throughout history, with the objective of reducing mistakes, errors, and adverse events in care. Florence Nightingale contributed considerably to patient safety by establishing principles of aseptic technique in care of wounded people during the Crimean War (1853–1856) (Rodríguez‐Herrera and Losardo 2018). Nightingale took notes on all care practices she observed, aiming to organize and systematize nursing care by using observation as a scientific method. By doing this, she documented information (which was made possible by her skills in mathematics and statistics) and showed that mortality was related mostly to precarious hygiene, a finding that sparked a revolution in military health and highlighted the importance of nursing interventions for decreasing the number of preventable deaths (Amezcua 2020).

With this milestone, Nightingale turned patient safety into a central aspect of nursing and emphasized that permanence, continuity, and contingency were fundamental factors to take into consideration in order to provide care free from threats to the physical integrity of individuals (Cometto et al. 2011).

It is important to clarify that Nightingale did not formulate the contemporary concept of patient safety, nor that of harm reduction as understood today. In this study, her writings are analyzed using contemporary patient safety frameworks to identify principles later systematized in modern safety theories.

Although Florence Nightingale has historically been recognized for her contributions to modern nursing, contemporary scholarship has increasingly problematized aspects of her legacy, particularly regarding colonialism, professional power structures, and the construction of nursing historiography. Although we acknowledge these controversial elements within her work, this study does not seek to revisit such broader debates but rather to reflect specifically on Nightingale's contributions to patient safety practices.

At present, the approach to patient safety is guided by systemic thinking, which recognizes that human beings are susceptible to mistakes and errors and that, in order to prevent them, it is necessary to create safe systems that identify failures before they cause losses. The widely applied model in this systemic logic is that of James Reason, who proposed the Theory of Active and Latent Failures (Reason 2000). The objective of this theory is to elucidate the processes that contribute to human error so this information can be applied in practice by creating mechanisms to contain harmful effects resulting from errors. Therefore, this theoretical perspective based on the understanding of failures aims to create barriers that can prevent losses from happening. In other words, the barriers reduce the chances that errors will occur and, if they do, that they will affect patients and cause harm.

Reflecting on Nightingale's legacy in the context of patient safety in the 21st century allows for an understanding that, even after 200 years, her knowledge and practices are still essential for the survival and safety of patients. The COVID‐19 pandemic highlighted the relevance of some of the principles Nightingale advocated, especially regarding the prevention of infection, which continues to pose a considerable threat to both communities and health professionals. The objective of the present article is to offer an analysis of Florence Nightingale's legacy based on a examination of how her principles correlate to contemporary patient safety ideas. By reviewing her practices and theories, we seek to understand how her approaches to infection prevention and health promotion remain relevant in scenarios with modern challenges. In this manner by taking into account the conceptual model of patient safety derived from James Reason's (2000) work we intend to shed light on the permanent importance of Nightingale's care model and propose reflections on its application and adaptation in the present day.

## 2 Background

Previous studies have explored Nightingale's legacy in terms of her innovative practices, which considerably improved care and patient outcomes. Nightingale rose to prominence during the Crimean War in the 1850s, during which she implemented rigorous hygiene techniques and proper sanitation, and organized patient care systems, which led to a remarkable decrease—from 42% to 2%—in the mortality rate of wounded soldiers (Ribeiro et al. 2022; Martini and Lippi 2021). These historical accounts have been extensively documented in biographical and analytical studies of her work (Baly 1986; Bostridge 2008).

Some authors writing about Nightingale's contributions have confirmed that, over the second half of the nineteenth century, Nightingale's ideas about environmental challenges and the importance of environmental health for disease prevention and control considerably influenced patient care and treatment processes. Her innovative ideas contributed to substantial changes in health practices and stressed the need for clean environments and proper conditions for patient recovery (Breigeiron et al. 2021; Kolagari 2023).

Other studies have confirmed that her care strategies, with an emphasis on cleaning, infection control, and patient well‐being, laid the foundations for the modern principles of nursing and the importance of patient safety (Baly 1986; McDonald 2010). Nightingale's data‐based approach, including statistical analysis and record keeping, contributed to advances in public health research and practices (Bostridge 2008; Ferreira et al. 2020; McDonald 2004; Nelson and Rafferty 2010). Her contributions to the development of statistics applied to health allowed more accurate analysis of care conditions and outcomes and promoted a more scientific, evidence‐based approach (Ribeiro et al. 2022; Martini and Lippi 2021; Baly 1986; Breigeiron et al. 2021).

In summary, studies have pointed out that Nightingale's work showed that simple but systematic measures could have an important impact on reducing the spread of diseases and improving global health outcomes. Nightingale's advocacy of patient well‐being led to the establishment of safer health practices, rigorous cleaning standards, and the recognition of the role played by nurses in ensuring patient safety (Ribeiro et al. 2022; Breigeiron et al. 2021; Nelson and Rafferty 2010; Emami Zeydi et al.^2021^).

Regarding current knowledge about patient safety, studies seek to formulate strategies to prevent incidents and adverse effects in health systems. These strategies are seen as safety barriers and take shape as devices, instruments, protocols, educational programs, and other health technologies focused on system safety. A body of evidence has confirmed the positive effects of developing and implementing these interventions to achieve various global safety goals, such as reductions in medication errors, falls, and pressure injuries, and improvements in communication during transitions of care (Benarroum Marín 2023; Reis 2019).

Additionally, analyses have also focused on the need to promote organizational cultures based on the commitment to values and beliefs that prioritize patient safety. This implies recognition of the importance of teamwork, engagement of leaders in providing safe healthcare, adoption of a proactive attitude in face of errors, open communication between professionals, encouragement of the notification and reporting of safety incidents, and effective organizational learning (Reis 2019). A positive safety culture is one of the essential requirements for reducing the occurrence of incidents, especially preventable adverse events. This can be accomplished by learning and redesigning processes.

The studies of Nightingale's lessons have indicated that her legacy still inspires efforts to make patient safety a priority, improve healthcare quality, and promote the well‐being of every individual (Santainés Borredá 2015). Consequently, analyzing how her principles have been reflected in current policies, guidelines, and concepts that support safety in health institutions could make an innovative and relevant contribution to our understanding of her legacy.

## 3 Inquiry Approach

This is a discursive article that explores Nightingale's legacy in the context of patient safety, based on the knowledge found in her book *Notes on Nursing: What It Is and What It Is Not*. The study was based on a close reading of Nightingale's book, with the aim of identifying key concepts and recommended practices related to patient safety. The book was examined in its entirety through critical and systematic reading of the pertinent sections, allowing for the identification and organization of concepts associated with Nightingale's environmental theory, particularly the notions of environment and patient.

These findings were subsequently analyzed through a cross‐referencing process that considered concepts and assumptions such as patient safety, systemic safety models, active and latent failures, safety barriers, and safety culture. This made it possible to understand the similarities and differences between the principles of both theoretical–conceptual frameworks. This study analyzes Florence Nightingale's legacy for patient safety 175 years after she developed her principles, and reflects on their consequences in the current context.

## 4 Nightingale's Principles Related to Patient Safety

Nightingale formulated her ideas after multiple trips to Europe and Egypt between 1849 and 1853 (Peres et al. 2021), during which she analyzed hospital reports and reviewed official publications related to public health, which allowed her to learn the art of caring (Núñez Carrasco 2011). She also gained experience during the Crimean War (World Health Organization 2021).

Nightingale emphasized that, in order to mitigate harm to patients, it is necessary to understand the effects the environment may impose on health and give priority to elements such as hygiene, cleanliness, and hospital ventilation and organization, which together constitute the basis of harm risk reduction (Cometto et al. 2011). Her principles of patient safety can be systematized into interrelated principles: pure external air, infection prevention, cleanliness in bed linens, and management with attention to small details.

The principle of pure external air is grounded in Nightingale's assertion that the patient must breathe air that is “as pure as the external air, without chilling him” (Nightingale 1989, 5). She emphasized that ventilation should draw air exclusively from the outside, ideally through windows positioned to capture the freshest air available (p. 5), and warned against the practice of keeping windows hermetically closed. According to Nightingale, such a practice exposes “the sick to all the dangers of an infected atmosphere, because she was afraid that, by admitting fresh air, the temperature of the ward would be too much lowered” (p. 7). In this sense, inadequate ventilation constitutes a direct risk to patient safety, as it increases exposure to environmental harm.

The principle of infection prevention is directly associated with environmental and nursing practice. Nightingale observed that maintaining open windows and covering patients lightly was associated with a reduction in infection, particularly in cases such as smallpox: “They have ventured to cover the patients lightly and to keep the window open; and we hear much less of the ‘infection’ of small‐pox than we used to” (p. 20). She affirmed that “true nursing ignores infection, except to prevent it” (p. 21), and indicated that “cleanliness and fresh air from open windows, with unremitting attention to the patient, are the only defense a true nurse either asks or needs” (p. 21). In this context, Nightingale further asserted that “wise and human management of the patient is the best safeguard against infection” (p. 21). This proposition establishes infection prevention as an outcome of environmental regulation and continuous nursing attention.

The principle of cleanliness in bed linens reflects Nightingale's understanding of the relationship between the patient's body and the environment. She argued that “feverishness is generally supposed to be a symptom of fever — in nine cases out of ten it is a symptom of bedding” (p. 63) and criticized nurses who considered only the patient, not the sick room, as their responsibility: “Nurses often do not think the sick room any business of theirs, but only the sick” (p. 66). Nightingale emphasized that “the nurse must be able to get easily to both sides of the bed, and to reach easily every part of the patient without stretching” (p. 64). She warned that placing a blanket under patients in situations with a risk of bed sores “retains damp and acts like a poultice” (p. 66). Nightingale also stated that “poisoning by the skin is no less certain than poisoning by the mouth — only it is slower in its operation” (p. 79), and highlighted that “washing, however, with a large quantity of water has quite other effects than those of mere cleanliness. The skin absorbs the water and becomes softer and more perspirable. To wash with soap and soft water is, therefore, desirable from other points of view than that of cleanliness” (p. 80). This proposition situates cleanliness in bed linens as determinants of patient safety through their impact on bodily integrity and exposure to harm.

The principle of management and attention to small details underpins all aspects of Nightingale's principles. She asserted that “all the results of good nursing, as detailed in these notes, may be spoiled or utterly negativated by one defect, viz.: in petty management” (p. 25) and emphasized that “circumstances must vary with each different case” (p. 25). Nightingale highlighted that many fatal outcome results from the absence of the nurse at a critical moment, noting that adverse events often occur “because ‘he’, or still oftener ‘she’, ‘was not there’“ (p. 27). She further observed that failures in management are more frequent in private houses than in institutions, where the consequences would be more evident and severe. This proposition highlights management as a central mechanism for preventing harm in patient care.

Nightingale's principles reveal a coherent framework for patient safety based on environmental control, infection prevention, cleanliness, and systematic management. They demonstrate that patient safety, as conceived by Nightingale, is inseparable from nursing practice and from the continuous assessment and control of harm risks inherent in the care environment.

## 5 Cross‐Reference Between Nightingale's Principles and Patient Safety Conceptual Foundations

The main premise of patient safety is the prevention and reduction of harm in healthcare. The concept is defined as a structure of organized activities that creates cultures, processes, procedures, behaviors, technologies, and environments that reduce risks steadily and sustainably, decrease the occurrence of preventable harm, make errors less likely, and lower the impact of harm when it happens (Travieso 2020). The objective of the systemic model for analyzing safety incidents is to understand their contributing factors by examining vulnerabilities in health systems, including both active factors related to professional practice and latent factors associated with organizational management. This knowledge is applied to inform new strategies to improve the several defense layers of the patient care process (Wiegmann et al. 2022). Therefore, the final goal is to prevent system vulnerabilities caused by failures—similar to holes in the many layers of Swiss cheese—from creating the conditions that bring risks of harm to patients. Such occurrences mean that all the defense layers were broken through. From this perspective, safety barriers come into play to prevent the failures from affecting patients (Wiegmann et al. 2022).

Nightingale made countless contributions to the professional practice of nursing, including the systematization of care. She revolutionized practice by implementing what can be recognized today as early forms of patient classification systems. By adopting infection prevention measures, giving priority to more serious cases, and organizing wards through the practical separation of patients according to disease severity and risk of contagion (Bostridge 2008), she raised the status of nursing and nursing care.

Nightingale also employed statistical methods to monitor clinical outcomes across different categories of cases, thereby enabling systematic comparisons and informed decision‐making (McDonald 2003). This form of systematization, which prioritized clinical severity over social status, laid the foundations for contemporary risk management and patient safety approaches (Baly 1986; Nelson and Rafferty 2010).

Building upon the conceptual framework presented in the previous section, the analysis of Nightingale's principles about patient safety reveals important similarities with contemporary patient safety frameworks. Her emphasis on infection prevention and cleanliness remains relevant today through infection prevention and control and biosafety protocols. Systematized actions, strongly suggested by Nightingale in her book, such as hand hygiene, room ventilation, environmental organization and cleanliness, infection rate monitoring, and staffing adequacy and commitment, find their counterparts in present‐day technological tools in the clinical and management spheres of healthcare to prevent errors and potential adverse events, including professionals updated by means of training and educational technologies, clinical protocols, and checklists, among others (Seshia et al. 2018). These technologies are the barriers that play a key role in health systems, since their function is to protect potential victims (Wiegmann et al. 2022). The principle is that every system that presents risks must have a defense mechanism. The various developed technologies can be seen as useful barriers for the defense of health systems (Wiegmann et al. 2022).

It could also be observed that managing small details regarding infrastructure, environmental conditions, and construction planning, especially the shape of hallways, was already part of Nightingale's range of concerns. She understood that inadequate infrastructure can directly influence patient safety and care effectiveness. In contemporary safety analyses, these elements are classified as latent factors, particularly as preconditions related to the physical environment, tools, and technologies (Wiegmann et al. 2022). Latent failures occur at higher levels in the system, above the unsafe acts level (active failures). This includes the organization, supervision, and precondition levels. These failures are called “latent” because, when they occur, they often go unnoticed. They can remain in the system for a long time before being identified (Wiegmann et al. 2022).

The environment and infrastructure conditions mentioned by Nightingale are latent factors classified as preconditions, that is, they are environmental factors, which include tools and technologies (design of equipment and control mechanisms, characteristics of displays and interfaces, layouts of checklists, task factors, and automation) and the physical environment (lighting, layout, noise, disorder, and workplace design) (Wiegmann et al. 2022). The combination of preexisting risk factors and latent conditions in health organizations can lead to physical, psychological, and material harm, among others (Reason 1990, 1997).

Another point of convergence lies in Nightingale's use of outcome evaluation. She had already assessed the effectiveness of proposed actions when she designed the polar area diagram, which presented a graphic, statistical representation of death rates resulting from hospital infections and showed that they decreased considerably after the actions she proposed were implemented. The type of assessment proposed by Nightingale culminated in the analysis of management practices by accreditors in the 21st century, when they seek to verify the presence of a patient safety culture by examining continuing education, management of the risks to patients, implementation of care protocols in order to minimize these risks, evaluation of implemented strategies aiming to propose adjustments, and cycles of improvement (World Health Organization 2021).

Therefore, by means of these principles, Nightingale established the early ideas of patient safety based on critical elements, such as monitoring practices (prevention of pure airborne infections), managing risk (management of and attention to small details), and environmental management (cleanliness in bed linens). These principles allowed professionals to understand that, from Nightingale's point of view, care delivered by nurses is guided by the restoration principle, understood here as the idea that nursing care facilitates the restoration of health through the intentional creation and maintenance of a proper care environment capable of facilitating patient recovery (Núñez Carrasco 2011).

Despite these similarities, an important difference emerges in how failures are conceptualized. Nightingale emphasized “carelessness” as the source of “failure,” which implies that nurses are responsible for it, starting from behaviors that emerge from not knowing about risk and harm. The system safety perspective does not include blaming the people involved in the occurrence of failures. Instead, the focus is on getting to know the characteristics behind their occurrence, aiming to avoid their repetition (Santainés Borredá 2015). This is a consequence of the understanding that making mistakes is human and that it is impossible to manage all human risk factors; therefore, there is an attempt to control them by implementing barriers that anticipate and prevent mistakes.

This historical attitude of holding nurses accountable for mistakes can be related to a punitive culture that still exists, in opposition to safety culture, which considers mistakes as a systemic process (Reason 2000, 1997). For this reason, it is important to consider the contextual complexity in which mistakes occur, which can be connected with latent conditions in health services (Reason 2000).

## 6 Patient Safety Nearly 200 Years After Nightingale

Despite Nightingale's emphasis on blame, she highlighted essential elements for the training of professionals, who must be able to perform their work with excellence, carrying out activities that involve skills and practices and can save more lives than many might imagine (Nightingale 1989). A comprehensive and integrated approach to care provided by nurses is fundamental to achieving this level of excellence and is a crucial factor for ensuring patient safety (Benarroum Marín 2023). In this respect, an important similarity can be identified between Nightingale's principles and contemporary patient safety frameworks, as both emphasize prevention, professional competence, and care quality as central elements of safe care.

This convergence highlights how her environmental and organizational principles paved the way for proposed innovations that have affected the understanding of what patient safety and care quality are to this day and remain aligned with current patient safety policies focused on risk reduction and harm prevention. In the following section, we revisit these contributions to offer a contemporary interpretation of their relevance in present‐day health systems.

The history of nursing since Nightingale has placed professionals in the position of being socially responsible for human safety, making the act of caring an essential activity. Based on her view of the role played by the women of her time and her perfectionism, Nightingale held nurses highly accountable for “processes or accidents.” Because of this way of conceptualizing nursing, the professional practice inherited behavior standards related to patient safety actions, such as hand hygiene, hospital infection control, and medication safety and administration. While these standards reinforced individual professional responsibility, they also laid the foundation for later patient safety approaches. Unlike Nightingale's emphasis on professional accountability, contemporary patient safety frameworks prioritize a systematic analysis of failures and the implementation of barriers to prevent error recurrence.

In contemporary times, concerns about patient safety have taken shape in the World Health Organization's Global Patient Safety Action Plan. Its vision is “a world in which no one is harmed in healthcare, and every patient receives safe and respectful care, every time, everywhere” (World Health Organization 2021, 8), and its mission is “to drive forward policies, strategies and actions, based on science, patient experience, system design and partnerships, to eliminate all sources of avoidable risk and harm to patients and health workers” (World Health Organization 2021, 8). The plan stresses the guiding principles of strengthening safety culture, such as: engaging patients and relatives in safe care; valuing collaborative work in order to achieve results; analyzing and sharing data in order to generate learning; translating evidence into useful and measurable improvements; having the nature of care inform policies and actions; using scientific knowledge and patient experience to improve safety; and investing in safety culture to design and provide healthcare (World Health Organization 2021).

Comparison of this 2021–2030 global plan with Nightingale's environmental principles shows how topical the latter are regarding patient safety. Some of these can be explored through analytical questions that emerge from this comparison: How much do health systems favor nursing care? What intervening factors are related to adverse events in nursing care? How can failures in nursing care be socially accepted from a systemic perspective? How can preventable adverse events be mitigated if the importance of nursing is not properly recognized by society and health systems? (World Health Organization 2021) Therefore, Nightingale's principles continue to be crucial foundations of patient safety, even 200 years after their creation. Additionally, her holistic approach, which considers the environment to be a fundamental part of patient recovery, has been translated into current patient‐centered care practices.

In patient‐centered care, professionals must be familiar with and respect patients' and relatives' preferences and wishes, and incorporate their clients' knowledge, beliefs, and cultural aspects into care planning; share clear, complete, and understandable information, which contributes to decision‐making; and encourage patients and relatives to participate, as active and critical partners, in the care process (Carman et al. 2013). From a safety perspective, active participation of patients in care is a strategy to prevent failures and adverse events. However, a key difference emerges in relation to patient and family engagement. The global plan stresses guiding principles such as engaging patients and relatives in safe care, valuing collaborative work, and using patient experience to improve safety (Carman et al. 2013). Engaging patients and relatives as active partners in safe care was not an explicit principle in Nightingale's principles, which focused primarily on nursing responsibility, environmental control, and professional vigilance. It should be pointed out that the current health reality is not comparable to that experienced by Nightingale, at least not to the described conditions of the Scutari Military Hospital, which were marked by filth, lack of ventilation, considerable mortality, and disorder (Morales Moreno and Herrera Justicia 2020; Attewell 2010).

The recent health context experienced during the COVID‐19 pandemic, in which care in health services became unsafe because of professionals' work overload, unclear task responsibilities, insufficient supervision, lack of knowledge about the disease, scarce or obsolete resources, and incorrect maintenance of facilities, among other factors, resulted in a high number of adverse events, which contributed to high rates of morbidity and mortality (Cometto et al. 2011; Seshia et al. 2018). This scenario highlights both similarities and differences between Nightingale's principles and contemporary patient safety frameworks. The unsafe care conditions observed during the pandemic closely mirror the environmental risks described by Nightingale in the Scutari Military Hospital, reinforcing the enduring relevance of her principles related to the environment, organization, and management. At the same time, the response to the pandemic relied on global coordination mechanisms, standardized protocols, and international patient safety policies that extended beyond the scope of Nightingale's historical context.

The impact of the pandemic on patients' and professionals' safety coincided with the announcement that 2020 would be the International Year of the Nurse and the Midwife to celebrate the 200th anniversary of Nightingale's birth. The year that was supposed to be celebrated with events and festivities was dominated by the challenges of dealing with this global health event, which tested nursing skills in multiple settings and affected the profession's visibility (Amezcua 2020; Salas‐Nicás et al. 2020).

This COVID pandemic situation was an opportunity for nurses, whose job is not always properly recognized in health services, to face this reality, which was totally adverse to safe care, with commitment and discipline. Occupational precariousness characterized nurses' work settings, salaries, and working conditions. But they also faced a lack of safety related to infection by a disease unknown to all; several studies have documented the lack of personal protective equipment at the beginning of the pandemic (Salas‐Nicás et al. 2020; Silva et al. 2021; Emami Zeydi et al. 2021).

Just like the nurses led by Nightingale during the Crimean War, 21st century nurses have been influenced by historical and temporal foundations of nursing practice, shaped by military traditions, religious values, and the women's heritage as caregivers (Alcántara and Santos 2022). In contexts like this, it is impossible to ensure the clinical safety of patient care, as shown by a study from the Madrid Health Service which described that, during the first three COVID‐19 waves, 36,494 incidents were reported, a number that was 60.7% lower before the pandemic (Macías Maroto et al. 2022). The important participation of nursing teams in the frontline health workforce during the COVID‐19 pandemic confirms what has been shown in different studies, which is that nurse are the healthcare professionals who play a fundamental role in promoting patients' health, exemplified by the application of the correct hand hygiene technique (including a higher frequency of the practice) to prevent infections (Reis 2019) and the implementation of steps to prevent medication errors (Manzo et al. 2019).

In Nightingale's historical context, her principles of hygiene, infection control, and the care environment, combined with the application of her statistical analysis methods and evidence examination protocols, were fundamental to steadily improving care quality. These are the cornerstones of modern nursing practices and safety protocols, which have allowed health institutions to monitor and respond to risk indicators. Contemporary patient safety policies expand these principles by explicitly incorporating patient engagement, systemic analysis of failures, and institutional safety culture, elements that were not central to Nightingale's original framework. This has had an impact on the guidance materials used by accreditation organizations in the present day, which contain checklist regarding structure, processes, and results, with the objective of determining whether the institution that provides the health services being evaluated offers safe and effective healthcare (Oliveira et al. 2020; World Health Organization 2017).

Therefore, Nightingale's principles about patient safety, as recorded in *Notes on Nursing*, have remained valid up to contemporary times. Many of the elements within the current World Health Organization's definition and guiding principles can be traced back to her guidance on how best to manage patient care.

## 7 Conclusion

Despite ongoing historiographical debates surrounding Florence Nightingale's broader legacy, her contributions to environmental care and patient safety remain historically significant and continue to influence contemporary healthcare practices. More than a century after her death, several principles discussed in her writings, including ventilation, hygiene, observation, organization of care, and prevention of harm, continue to resonate within patient safety frameworks.

Nightingale advanced perspectives that may be associated with systemic thinking in healthcare, including professional accountability for the care provided, analyzing the causes of discontinuity of care quality, adopting a critical perspective to implement changes, and emphasizing the ethical principle of avoiding harm to patients. Her experience in coordinating and innovating also contributed to the organization and management of nursing practices in institutional settings. Although important challenges persist in implementing patient safety and quality‐of‐care measures in contemporary health systems, Nightingale's reflections on the care environment and professional responsibility continue to offer relevant historical contributions to discussions on safe and effective healthcare practices.

## Funding

The authors have nothing to report.

## Ethics Statement

The authors have nothing to report.

## Conflicts of Interest

The authors declare no conflicts of interest.

## AI Disclosure Statement

The authors used generative artificial intelligence (AI) tools, including ChatGPT (OpenAI) and Grammarly for language editing, such as improving grammar, clarity, and sentence structure. These tools were not used for the conceptualization of the study, data collection, data analysis, interpretation of findings, or development of scientific conclusions. All intellectual content, arguments, interpretations, and final revisions remain the responsibility of the authors.

## Permission to Reproduce Material

Not applicable.

## Data Availability

Data sharing not applicable to this article as no datasets were generated or analyzed during the current study.
